# Normal variations in personality predict eating behavior, oral health, and partial syndrome bulimia nervosa in adolescent girls

**DOI:** 10.1002/fsn3.1425

**Published:** 2020-01-27

**Authors:** Mark S. Allen, Davina A. Robson, Sylvain Laborde

**Affiliations:** ^1^ School of Psychology University of Wollongong Wollongong NSW Australia; ^2^ German Sport University Cologne Koln Germany

**Keywords:** body weight, diet, eating disorder, five‐factor model, temperament

## Abstract

Eating disorders are among the most prevalent disorders in adolescence and can have negative consequences including poor quality of life, medical complications, and even death. This study addresses whether normal variations in personality relate to eating behavior and eating disorder symptomatology in adolescent girls. Participants were a near‐representative sample of Australian adolescent girls (*n* = 1,676). Three personality traits (neuroticism, extraversion, and conscientiousness) were assessed at age 12 and again at age 14, and self‐reported eating and weight management behaviors were assessed at age 14. After controlling for sociodemographic factors, higher levels of conscientiousness at age 12, and increases in conscientiousness between ages 12 and 14, were associated with greater fruit and vegetable consumption, a lower intake of high fat foods and high sugar drinks, less frequent meal skipping, better oral health, and decreased risk of partial syndrome bulimia nervosa at age 14. Higher neuroticism at age 12 was associated with more frequent meal skipping, and increases in neuroticism between ages 12 and 14 were associated with more frequent meal skipping and increased risk of partial syndrome bulimia nervosa at age 14. Extraversion was generally unrelated to eating and weight management behaviors. These findings provide evidence that normal variations in personality are related to eating behavior, oral health, and eating disorder symptoms during midadolescence.

## Graphical abstract

Personality at age 12 and change in personality between age 12 and age 14 predict eating behaviors (high fat foods and high sugar drinks), oral health, and eating disorder symptoms at age 14.

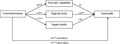

## 1 INTRODUCTION

Body weight is an important public health issue that has implications for serious and chronic health conditions. Rates of being overweight or obese are increasing in industrialized nations (Abarca‐Gómez et al., 2017; Ng et al., 2014) and accounted for about four million deaths and 120 million disability‐adjusted life years worldwide in 2015 (GBD 2015 Obesity Collaborators, 2017). Despite the increasing rates of overweight and obesity, thin and ultrathin bodies are often idealized in modern culture, particularly among girls and young women (Grogan, 2016). This incredibly thin body shape is unattainable for the average person and can lead to body dissatisfaction and unhealthy eating behaviors (Stice & Whitenton, 2002; Walker, White, & Srinivasan, 2018). Eating disorders—characterized by abnormal eating behaviors and psychological disturbance related to food and weight—are the third most common chronic illness among adolescents (after obesity and asthma) and can lead to poor quality of life, medical complications, and even death (Agh et al., 2016; Arcelus, Mitchell, Wales, & Nielsen, 2011). Eating disorders are more common among girls than boys (~85% of those diagnosed are girls; Smink, van Hoeken, Oldehinkel, & Hoek, 2014) with the peak age of onset being 14–19 years (Herpertz‐Dahlmann, 2015). This study addresses whether normal variations in personality predict eating behavior and eating disorder symptomatology in adolescent girls.

Much research has explored associations between personality traits and body weight across the life span (Allen & Vella, 2016; Sutin, Ferrucci, Zonderman, & Terracciano, 2011; Sutin, Rogers, et al., 2015a). This research has found that low conscientiousness and high neuroticism are most important for excess body weight. New research has also explored how personality traits relate to being underweight in adulthood (Allen, Vella, Swann, & Laborde, 2018; Sutin & Terracciano, 2016). These studies found that adults categorized as underweight (BMI < 18.5) were more introverted compared with normal weight (BMI = 18.5–25.0) and overweight (BMI > 25.0) persons. This is a surprising finding given that extraverted individuals are generally more physically active (Allen & Laborde, 2014; Sutin et al., 2016), and an active lifestyle is associated with a lower BMI (Kimm et al., 2005). This counterintuitive finding might be explained by eating behavior. Introverted individuals might be less inclined to take part in physical activity but might also be more susceptible to body image concerns that can lead to low body weight.

There is now good evidence that people with higher levels of neuroticism, and lower levels of extraversion and conscientiousness are at greater risk of thin‐ideal internalization and negative body image (Allen & Walter, 2016; Martin & Racine, 2017). This is important as body image concerns are known to affect eating habits and increase risk of disordered eating behavior (Walker et al., 2018). Researchers have started to explore how personality traits relate to consumption of particular foods and beverages. Conscientiousness, and to a lesser extent extraversion, appear to be the most important personality dimensions for eating behavior (Lunn, Nowson, Worsley, & Torres, 2014; Mõttus et al., 2013, 2012; Olsen, Tuu, Honkanen, & Verplanken, 2015). Research has found that higher levels of extraversion (Conner et al., 2017; Keller & Siegrist, 2015) and conscientiousness (Allen, Vella, & Laborde, 2015; Conner et al., 2017) relate to higher fruit and vegetable consumption, whereas neuroticism is unrelated to fruit and vegetable consumption (Allen et al., 2015; Conner et al., 2017; Keller & Siegrist, 2015). There is also some emerging evidence that higher levels of extraversion (Keller & Siegrist, 2015) and neuroticism (Tiainen et al., 2013) relate to a greater consumption of high sugar drinks. One study also explored meal skipping behavior in Ghanaian university students and found that personality traits were generally unrelated to meal skipping (Intiful, Oddam, Kretchy, & Quampah, 2019).

Because dietary habits are important for oral health (Salas et al., 2015), researchers have explored further whether personality relates to oral health. There is some emerging evidence that higher levels of extraversion and conscientiousness, and lower levels of neuroticism relate to better self‐reported oral health (Al‐Omiri, Alhijawi, Al‐Shayyab, Kielbassa, & Lynch, 2019; Montero et al., 2016; Thomson, Caspi, Poulton, Moffitt, & Broadbent, 2011) and oral‐health related quality of life (Clijmans, Lemiere, Fieuws, & Willems, 2015; Takeshita et al., 2015) in adulthood. Furthermore, because personality is important for body image (Allen & Walter, 2016; Martin & Racine, 2017) and associated eating behaviors (Walker et al., 2018), researchers have explored whether personality traits relate to risk of eating disorders (Atiye, Miettunen, & Raevuori‐Helkamaa, 2015; Cassin & von Ranson, 2005). Research synthesis is difficult given the varying conceptualizations of personality used across studies, but in general, this research has found that high levels of neuroticism, and to a lesser extent low levels of conscientiousness, relate to an increased risk of having ever been diagnosed with an eating disorder (Bogg & Roberts, 2004; Cassin & von Ranson, 2005; Marzola, Fassino, Amianto, & Abbate‐Daga, 2017).

### 1.1 The current study

Personality appears to have an important role in eating and weight management, and three personality traits—neuroticism, extraversion, and conscientiousness—stand out as important correlates of diet, oral health, and disordered eating behavior. However, further research is needed for a number of reasons. First, most research has explored associations in adult samples (see Lunn et al., 2014) and how personality relates to eating behaviors in early and midadolescence is less well understood. Second, most research has been cross‐sectional in nature (Cassin & von Ranson, 2005; Lunn et al., 2014) and while research has explored how personality relates to change in eating behavior over time (Allen et al., 2015), whether personality change is important for diet and disordered eating behavior remains unknown. Research has shown that personality is more unstable during adolescence than at any other life point (Roberts, Walton, & Viechtbauer, 2006) and short‐term personality change might be particularly important for health behaviors at this critical developmental stage. Moreover, adolescents who increase in levels of conscientiousness (increase their desire to do well and take obligations more seriously) and extraversion (become more confident and motivated by status), and decrease in levels of neuroticism (become less anxious and self‐conscious) might become less interested in body image and show better dietary and oral health habits.

The research conducted to date shows that neuroticism, conscientiousness, and extraversion are important for dietary habits, oral health, and eating disorder diagnosis in adult samples and further research is needed to establish the importance of personality traits for each of these outcomes in adolescent samples. The current research sought to test (in a representative sample of Australian girls) whether personality traits at age 12, and personality trait change between ages 12 and age 14 are associated with eating behavior (diet), frequency of meal skipping, oral health, and eating disorder symptomatology at age 14—the age when adolescent girls start to become susceptible to thin‐ideal internalization and disordered eating (Herpertz‐Dahlmann, 2015). Based on past research in adults samples, we hypothesized that lower levels of neuroticism and higher levels of conscientiousness and extraversion at age 12, and decreases in neuroticism and increases in conscientiousness and extraversion between ages 12 and 14 would be associated with higher fruit and vegetable consumption, a lower intake of high fat foods and high sugar drinks, better oral health, less frequent meal skipping, and a decreased likelihood of eating disorder symptomatology.

## 2 METHOD

### 2.1 Sample

This study uses data from the Longitudinal Study of Australian Children (LSAC) Kindergarten (K) cohort. LSAC is a biennial panel survey launched in 2003 that aims to investigate child and adolescent social, economical, and cultural environments as they relate to adjustment and well‐being. This study uses data collected at wave 5 (Time 1) and wave 6 (Time 2). LSAC collects data from both adolescents and their parents. Only adolescent girls were used in this study given the low frequency of eating disorders among adolescent boys (O’Connor, Warren, & Daraganova, 2018; Smink et al., 2014). At Time 1, there were 1936 adolescent girls, with 1676 returning at Time 2. Attrition analyses show that, compared with those who returned, dropouts had a lower neighborhood socioeconomic position, *t*(1,931) = 2.80, *p *= .005, *d* = 0.13, and lower levels of conscientiousness, *t*(1,881) = 3.07, *p *= .002, *d* = 0.14. Only participants who returned at Time 2 were included in the study. Adolescent girls were 12.4 (±0.5) years at Time 1 and 14.4 (±0.5) years at Time 2. LSAC received ethical approval from the Australian Institute of Family Studies Ethics Committee.

### 2.2 Measures

#### 2.2.1 Personality

Parent‐reported adolescent personality traits were measured at Time 1 and Time 2 using the school‐aged temperament inventory (McClowry, 1995). This questionnaire assesses three personality dimensions that correspond to conceptualizations of *neuroticism* (e.g., “reacts strongly to disappointment”), *extraversion* (e.g., “uncomfortable at someone else's home for the first time”), and *conscientiousness* (e.g., “remembers homework without reminders”) in adults. These dimensions are often labeled “reactivity/emotionality,” “introversion/sociability,” and “persistence/perseverance” in younger samples, but are conceptually interchangeable (see McCrae et al., 2000). The parent‐report version of the school‐aged temperament inventory has demonstrated evidence of interitem reliability and predictive validity (McClowry, Halverson, & Sanson, 2003) but has not been subjected to critical tests of construct validity.

#### 2.2.2 Eating behavior

Fruit and vegetables, high fat foods, and high sugar drinks consumed the previous day were used as an index of adolescent eating habits at Time 2 (self‐report). *Fruit and vegetable intake* was assessed as the sum of three items: “Thinking about yesterday, how often did you have….”: (1) “fresh fruit?”, (2) “cooked vegetables?”, and (3) “raw vegetables or salad?”. Intake of *high fat food* was assessed as the sum of four items: “Thinking about yesterday, how often did you have….” (1) “meat pie, hamburger, hot dog, sausage or sausage roll?”, (2) “hot chips or French fries?”, (3) “potato chips or savory snacks”, and (4) “biscuits, doughnuts, cake or chocolate?”. Intake of *high sugar drinks* was assessed as the sum of two items: “Thinking about yesterday, how often did you have….” (1) “fruit juice?” and (2) “soft drink or cordial [not diet soft drink or diet cordial]?”. Responses to all items were scored as 0 (*not at all*), 1 (*once*), or 2 (*twice or more*). These items were developed purposefully for LSAC, based on questions used in previous cohort studies (Rutishauser, Webb, Abraham, & Allsopp, 2001). The 24‐hr recall approach to measuring dietary habits provides a useful proxy for typical eating behavior in children and helps avoid problems associated with self‐report assessments over longer time periods such as memory recall bias and social desirability (Johnson, 2002).

#### 2.2.3 Meal skipping

At Time 2, self‐reported frequency of skipping meals (other than breakfast) was assessed using a single item: “Do you skip meals other than breakfast because you are watching your weight?”, adapted from the adolescent dieting scale (Patton et al., 1997). Responses were scored as 1 (*seldom/never*), 2 (*sometimes*), 3 (*often*), or 4 (*almost always/always*). The adolescent dieting scale has demonstrated evidence of predictive validity in adolescent samples (Hinchliff, Kelly, Chan, Patton, & Williams, 2016).

#### 2.2.4 Oral health

At Time 2, self‐reported oral health was assessed using the PedsQ oral health scale (Steele, Steele, & Varni, 2009). Adolescents were asked: “In the past 1 month, how much of a problem has this been for you…” followed by five items: (1) “I have tooth pain,” (2) “I have tooth pain when I eat or drink something hot, cold, or sweet,” (3) “I have teeth that are dark in colour,” (4) “I have gum pain,” and (5) “I have blood on my toothbrush after brushing my teeth”. Each item was scored as 0 (*never*), 1 (*almost never*), 2 (*sometimes*), 3 (*often*), or 4 (*almost always*). Scores were summed, reverse coded, and recoded to a score between 0 and 100, with higher scores representing better oral health. The construct, criterion, and convergent validity of the PedsQ oral health scale has been supported in adolescent samples (Steele et al., 2009; also see Zaror et al., 2019).

#### 2.2.5 Partial syndrome bulimia and anorexia nervosa

The branched eating disorders test (Selzer, Hamill, Bowes, & Patton, 1996) was used to assess criteria for eating disorders according to the fifth edition of the diagnostic and statistical manual of mental disorders (DSM‐5; American Psychiatric Association, 2013). The test is designed for use in adolescent samples and identifies symptoms of eating disorders within the previous 3 months. Adolescents were classified as having *partial syndrome bulimia nervosa* if they met two of the following three diagnostic criteria: (1) reported their weight as being “very important” to how they feel about themselves, (2) reported that they had lost control of their eating, or had eaten far too much, at least weekly for at least 3 months, and (3) reporting at least one of the following behaviors in the last 3 months: (a) making themselves vomit as a means to control weight at least once per week, (b) taking tablets or medicines to control their weight at least once per week, (c) going all day without eating to control their weight on four or more days per week, or (d) exercising to control weight six or seven days a week for two or more hours.

Adolescents were classified as having *partial syndrome anorexia nervosa* if they met two of the following three diagnostic criteria: (1) being assessed as having a very low body weight sourced from their body mass index (BMI), (2) reported feeling afraid of gaining weight on two to three days a week or more; reporting being “very” or “extremely” concerned about gaining weight; as well as being assessed as having a lower BMI than normal, (3) reported their weight as being “very important” to how they feel about themselves; reported themselves as being “somewhat overweight” or “very overweight” and were assessed as having a lower BMI than normal, and (4) reported not having had their period in the last 3 months and not being pregnant at the time of interview (girls who had started menstruating only). In total, 55 girls (3.3% of sample) were classified as having partial syndrome bulimia or anorexia nervosa (partial syndrome bulimia nervosa, *n* = 49; partial syndrome anorexia nervosa, *n* = 6).

#### 2.2.6 Control variables

Parents provided information on indigenous status, home postcode, household income (in AUD per week), and number of people in the household at Time 1. Postcode and household income were used to provide two indirect estimates of socioeconomic status: neighborhood socioeconomic position (NSP) and adjusted household income. Using participants' home postcode, an estimate of NSP was determined according to the index of relative socioeconomic disadvantage (Australian Bureau of Statistics, 2008). Household income was standardized to the household size by dividing estimates by the square root of the number of people in the house (Australian Bureau of Statistics, 2005). Indigenous status was coded as 0 (*non‐aboriginal or Torres Strait Islander*) or 1 (*aboriginal or Torres Strait Islander*).

### 2.3 Data analysis

Linear regression models were used to explore whether personality at Time 1 and mean‐level change in personality (Time 2 score minus Time 1 score) were associated with fruit and vegetable consumption, intake of high fat foods, consumption of sugary drinks, frequency of meal skipping, and oral health at Time 2. Indigenous status, household income, and NSP were entered as predictors at Step 1, with Time 1 personality dimensions added at Step 2, and change scores added at Step 3. For partial syndrome bulimia nervosa, a binary logistic regression model was used in place of a linear regression model. Partial syndrome anorexia nervosa was not tested given the low number of adolescent girls coded as showing these symptoms (*n* = 6) and the corresponding low statistical power. There were missing data for some participants (<2% of cells were empty) that were handled using multiple imputation (Rubin, 1987). Across linear regression models, there was no evidence of multicollinearity (all VIF values < 1.8) and between zero and two potential multivariate outliers were observed in each model (Cook's distance values >0.02). Regression models were rerun with these potential outliers removed with no meaningful change to results, and we present findings for the full data sample. Statistical significance was set at 0.05 for all statistical tests.

## 3 RESULTS

**Figure 1:**
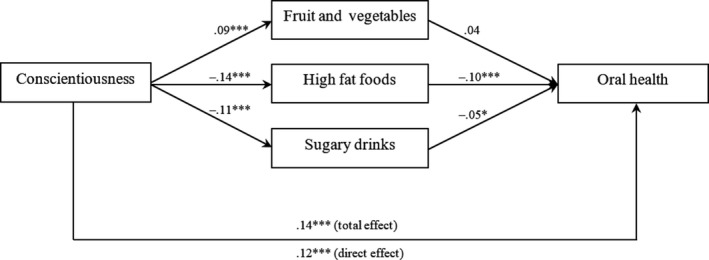
Multiple mediation model for oral health regressed on conscientiousness through diet variables (standardized coefficients reported; household income, socioeconomic position, and indigenous status held constant in model; computed using 10,000 bootstrap resamples)

Cross‐sectional and cross‐time zero‐order correlations are reported in Table 1, and findings from the regression models are reported in Table 2. There were significant effects at Step 1 (sociodemographic factors), Step 2 (baseline personality traits), and Step 3 (personality trait change) in all six regression models, with one exception (see Table 2). Higher levels of conscientiousness at age 12 were associated with a greater intake of fruit and vegetables, *b *= 0.20 (95% CI: 0.09, 0.31), a lower intake of high fat foods, *b *= −0.23 (95% CI: −0.33, −0.14), a lower intake of high sugar drinks, *b *= −0.16 (95% CI: −0.23, −0.09), and better oral health, *b* = 2.11 (95% CI: 1.34, 2.89) at age 14. Increases in conscientiousness between age 12 and age 14 were also associated with a greater intake of fruit and vegetables, *b *= 0.21 (95% CI: 0.08, 0.35), and a lower intake of high sugar drinks, *b *= −0.15 (95% CI: −0.24, −0.06), and a decrease in neuroticism between ages 12 and 14 was also associated with better oral health, *b *= −1.52 (95% CI: −2.48, −0.56), at age 14. Because conscientiousness was associated with both diet and oral health, we tested for a potential mediation effect. A multiple mediation model was run using standard procedures for ordinary least squares regression (Hayes, 2018). There was a significant mediation effect (*β *= 0.023 [95% CI: 0.013, 0.034]), with high fat food intake (*β *= 0.013 [95% CI: 0.006, 0.022]) and high sugar drinks intake (*β *= 0.006 [95% CI: 0.001, 0.013]) contributing significantly to the mediation model (Figure 1). In total, 16.3% of the association between conscientiousness and oral health could be explained by diet variables.

**Table 1:** Means, standard deviations, and bivariate correlations among measured variables

	*M*	*SD*	2.	3.	4.	5.	6.	7.	8.	9.	10.	11.	12.	13.	14.	15.
Time 1																
1. Indigenous status	1.7% (aboriginal)		−0.05	−0.10	0.04	0.00	−0.08	−0.01	0.06	0.04	−0.01	−0.06	−0.02	0.04	−0.01	−0.08
2. Household income (adjusted)	2,428.02	1,736.37		0.32	−0.06	0.02	0.13	0.07	−0.09	−0.12	−0.06	0.08	0.00	−0.07	0.01	0.15
3. NSP	1,012.85	66.99			−0.04	0.02	0.08	0.06	−0.07	−0.15	−0.07	0.10	−0.02	−0.04	0.00	0.11
4. Neuroticism	2.40	0.76				−0.17	−0.36	−0.03	0.09	0.04	0.11	−0.06	0.07	0.68	−0.12	−0.31
5. Extraversion	3.28	0.76					0.08	0.04	−0.02	−0.01	0.03	−0.01	−0.03	−0.16	0.68	0.04
6. Conscientiousness	3.81	0.75						0.10	−0.15	−0.13	−0.13	0.16	−0.08	−0.29	0.10	0.64
Time 2																
7. Fruit and vegetable intake	3.27	1.62							−0.04	−0.08	−0.06	0.07	−0.01	−0.04	0.05	0.13
8. High fat food intake	1.81	1.44								0.34	−0.03	−0.14	−0.03	0.10	−0.03	−0.14
9. High sugar drink intake	1.15	1.08									0.01	−0.12	−0.03	0.06	0.02	−0.16
10. Frequency of skipping meals	1.33	0.70										−0.11	0.29	0.14	0.00	−0.20
11. Oral health	87.95	11.45											0.01	−0.11	−0.02	0.15
12. Partial syndrome bulimia nervosa	2.9% (yes)													0.10	0.00	−0.11
13. Neuroticism	2.37	0.80													−0.19	−0.38
14. Extraversion	3.21	0.80														0.08
15. Conscientiousness	3.87	0.78														

Note

NSP, neighborhood socioeconomic position. Household income in AUD standardized to household size. Correlations greater than (or equal to) ± 0.05 are significant at the 0.05 level.

**Table 2:** Linear and logistic regression models for eating behavior and related constructs regressed on personality traits and covariates

	Fruit and vegetables	High fat foods	High sugar drinks	Oral health	Meal skipping	PS Bulimia Nervosa
Step 1	[Δ*F*_(3,1672)_ = 3.76]*	[Δ*F*_(3,1672)_ = 7.47]***	[Δ*F*_(3,1672)_ = 17.09]***	[Δ*F*_(3,1672)_ = 8.59]***	[Δ*F*_(3,1672)_ = 3.80]*	[$\Delta \chi_{\left(3,1672\right)}^{2}$ = 0.99]
Indigenous status	0.00	0.05*	0.03	−0.05*	−0.02	–
NSP	0.05	−0.04	−0.13***	0.08**	−0.06*	1.00 (0.98, 1.02)
Household income (adjusted)	0.06*	−0.08**	−0.08**	0.05	−0.05	0.79 (0.51, 1.21)
Step 2	[Δ*F*_(3,1669)_ = 5.52]**	[Δ*F*_(3,1669)_ = 11.31]***	[Δ*F*_(3,1669)_ = 6.48]***	[Δ*F*_(3,1669)_ = 11.54]***	[Δ*F*_(3,1669)_ = 12.73]***	[$\Delta \chi_{\left(3,1669\right)}^{2}$ = 12.22]**
T1 Neuroticism	0.01	0.04	−0.01	−0.01	0.08**	1.37 (0.94, 1.99)
T1 Extraversion	0.04	−0.01	0.01	−0.03	0.06*	0.89 (0.62, 1.29)
T1 Conscientiousness	0.09***	−0.12***	−0.11***	0.14***	−0.10***	0.66 (0.45, 0.95)*
Step 3a	[Δ*F*_(3,1666)_ = 3.83]**	[Δ*F*_(3,1666)_ = 1.72]	[Δ*F*_(3,1666)_ = 5.41]**	[Δ*F*_(3,1666)_ = 5.40]**	[Δ*F*_(3,1666)_ = 12.81]***	[$\Delta \chi_{\left(3,1666\right)}^{2}$ = 14.56]**
Δ Neuroticism	0.00	0.03	0.03	−0.08**	0.06*	1.67 (1.06, 2.63)*
Δ Extraversion	0.02	0.00	0.04	−0.04	−0.01	1.50 (0.92, 2.45)
Δ Conscientiousness	0.09**	0.05	−0.09**	0.04	−0.14***	0.62 (0.39, 0.98)*

Note

NSP, neighborhood socioeconomic position. Household income in AUD standardized to household size. T1, Time 1; Δ = change score (Time 2 score minus Time 1 score). Standardized beta coefficients reported for linear regression models, odds ratios and 95% confidence intervals reported for the logistic regression model. Indigenous status is automatically deleted from the logistic regression model as no adolescents coded as aboriginal Australian were diagnosed with partial syndrome bulimia nervosa.

* *p *< .05.

** *p *< .01.

*** *p *< .001.

Higher levels of neuroticism, *b *= 0.08 (95% CI: 0.03, 0.12), and extraversion, *b *= 0.05 (95% CI: 0.01, 0.10), and lower levels of conscientiousness, *b *= −0.09 (95% CI: −0.14, −0.04), at age 12 were associated with a more frequent occurrence of meal skipping at age 14. Increases in neuroticism, *b *= 0.07 (95% CI: 0.01, 0.12), and decreases in conscientiousness, *b *= −0.14 (95% CI: −0.20, −0.09), between age 12 and age 14, were also associated with more frequent meal skipping at age 14. Findings from the logistic regression model showed that lower levels of conscientiousness at age 12, OR = 0.66 (95% CI: 0.45, 0.95), increases in neuroticism between ages 12 and 14, OR = 1.67 (95% CI: 1.06, 2.63), and decreases in conscientiousness between ages 12 and 14, OR = 0.62 (95% CI: 0.39, 0.98), were associated with an increased likelihood of partial syndrome bulimia nervosa at age 14.

## 4 DISCUSSION

This study tested whether normal variations in personality relate to eating behavior and eating disorder symptomatology in adolescent girls. Results showed that higher levels of conscientiousness at age 12, and increases in conscientiousness between ages 12 and 14 were associated with higher fruit and vegetable consumption, a lower intake of high fat foods and high sugar drinks, less frequent meal skipping, better oral health, and decreased risk of partial syndrome bulimia nervosa at age 14. To a lesser extent, higher neuroticism at age 12 was associated with more frequent meal skipping, and increases in neuroticism between ages 12 and 14 were associated with more frequent meal skipping and increased risk of partial syndrome bulimia nervosa at age 14. The association between conscientiousness at age 12 and oral health at age 14 was mediated by healthy eating (high sugar drinks and high fat foods). Overall, conscientiousness stands out as most important for girls' weight management behavior in midadolescence.

The finding that conscientiousness is the most important personality trait for fruit and vegetable consumption is consistent with findings observed in adult samples (Allen et al., 2015; Conner et al., 2017; Keller & Siegrist, 2015). However, the current study does not support previous work that also found a small positive association between extraversion and fruit and vegetable intake (Conner et al., 2017; Keller & Siegrist, 2015). An important new finding was that personality trait change over 2 years related to healthy eating. Personality is most unstable during adolescence (Roberts et al., 2006) and the current research provides some initial evidence that adolescent girls who increase in levels of conscientiousness between ages 12 and 14 have more healthy eating habits at age 14 (higher intake of fruit and vegetables, and lower intake of high sugar drinks). People with high levels of conscientiousness have high self‐control, are less impulsive, are more responsible, and are better at delaying gratification (Roberts, Lejuez, Krueger, Richards, & Hill, 2014). It therefore follows that girls with greater increases in conscientiousness during this developmental stage show healthier eating behavior.

The finding that conscientiousness, but not extraversion or neuroticism, related to consumption of high fat foods and high sugar drinks, differs somewhat from previous work that found small effects for extraversion (Keller & Siegrist, 2015) and neuroticism (Tiainen et al., 2013). These differences in study findings could reflect differences in meal consumption autonomy between adults and adolescents. Adolescent girls often have less control over their fruit and vegetable intake that is also governed (somewhat) by parental influences. It might be the case that as adolescents become older, and gain more autonomy, extraversion and neuroticism become more important factors governing healthy eating. In addition to eating behavior, the study also found a positive association between conscientiousness and subsequent oral health and this is consistent with previous research observed in adult samples (Al‐Omiri et al., 2019; Montero et al., 2016; Thomson et al., 2011). However, the current research does not support findings from these studies that show high levels of extraversion and low levels of neuroticism relate to better oral health. The current research did, however, find that change in neuroticism was important for oral health as adolescents who increased in neuroticism between ages 12 and 14 reported poorer oral health at age 14. Because conscientiousness was related to both eating behavior and oral health, we explored whether eating behavior functioned as a mediator in the association between conscientiousness and subsequent oral health. We found that some of the variance (about 16%) connecting conscientiousness at age 12 to oral health at age 14 was shared with eating behavior. Given the rather small amount of shared variance, it is likely that other unknown factors are also important in connecting conscientiousness to oral health (e.g., frequency of flossing and length of time brushing teeth).

That meal skipping for weight management was associated with all three personality traits—higher neuroticism and extraversion, and lower conscientiousness—is an important new finding. A previous study explored these associations in a small sample of adults and found that these personality traits were unrelated to meal skipping (Intiful et al., 2019). In addition to measures at age 12, increases in neuroticism and decreases in conscientiousness between ages 12 and 14 were also associated with a greater occurrence of meal skipping for weight management. Findings for meal skipping did not mirror those for other eating‐related variables, but were similar to those for partial syndrome bulimia nervosa—indicating that meal skipping for weight management might be symptomatic of an eating disorder. Overall, 49 girls showed symptoms of bulimia nervosa (2.9% of sample). Despite the small sample size, the data indicated that girls with lower levels of conscientiousness, decreases in conscientiousness between ages 12 and 14, and increases in neuroticism between ages 12 and 14 were at greater risk of partial syndrome bulimia nervosa. This finding complements previous research that has found cross‐sectional associations between personality disorders and disordered eating in adolescence (Lilenfeld, Wonderlich, Riso, Crosby, & Mitchell, 2006) and associations between major personality dimensions and disordered eating in adulthood (Cassin & von Ranson, 2005). However, confidence intervals neared zero, and therefore, findings must be considered with some caution.

Strengths of this study include the large near‐representative sample of adolescent girls, the use of DSM‐5 criteria to establish eating disorder symptomatology, and testing mean‐level personality trait change in early adolescence. Nevertheless, there are some important limitations that readers must consider when interpreting study findings. First, the study used parent‐report measures of personality that provide only an approximation of adolescent personality traits (Laidra, Allik, Harro, Merenäkk, & Harro, 2006). Self‐report and parent‐report measures are subject to different biases, and future research might adopt multiple raters and triangulation to more accurately capture adolescent personality and personality trait change over time. Second, findings from the current research cannot be generalized beyond the current population of Australian girls. In contrast to what is often found in Western samples, conscientiousness is mostly unrelated to adiposity in Asian samples (Sutin, Stephan, et al., 2015b), and whether the current findings transfer to other world regions remains unknown.

A third potential limitation is that the current study does not include an assessment of autonomy over eating behavior. Personality might be more important in households where parents give adolescents more autonomy over their eating, and future research should explore parental influence over adolescent eating as a potential moderator. Finally, the current study is observational in nature and does not provide information on cause and effect. Important demographic factors were held constant in regression models, and personality was identified as an important independent correlate. However, it might be the case that poor diet, oral health, and eating disorder symptomatology contribute to personality change. Indeed, fruit and vegetable consumption has been found to predict change in personality over time (Allen et al., 2015) and oral health and eating disorder symptoms are important for quality of life (Sischo & Broder, 2011) that can contribute to stability and change in personality (Bleidorn, Hopwood, & Lucas, 2018). Experimental work is needed to identify whether personality‐based interventions can improve eating behavior and reduce risk of eating disorder symptoms in adolescent girls.

To conclude, this study has found that conscientiousness and (to a lesser extent) neuroticism are important for outcomes including dietary intake, meal skipping, oral health, and risk of partial syndrome bulimia nervosa in adolescent girls. Baseline personality levels and personality trait change were both associated with these outcomes, and eating behavior was found to mediate the association between conscientiousness and oral health. Findings from this study might be of interest to health practitioners working with eating disorder patients. Monitoring of personality trait change, along with body weight, might be a useful approach to early identification of those at risk of an eating disorder, as well as identification of those who might benefit most from inclusion in interventions that target risk reduction for eating disorders. We recommend further observational studies that test personality change over a longer time period and studies that consider important untested moderators such as eating autonomy (in alternative populations and cultures) to help establish the generalizability of findings observed in the current study.

## CONFLICT OF INTEREST

The authors declare no conflicts of interest.

## ETHICAL APPROVAL

LSAC received ethical approval from the Australian Institute of Family Studies Ethics Committee and the study conforms to the Declaration of Helsinki and European Medicines Agency Guidelines for human participants.
